# The specific DNA barcodes based on chloroplast genes for species identification of *Orchidaceae* plants

**DOI:** 10.1038/s41598-021-81087-w

**Published:** 2021-01-14

**Authors:** Huili Li, Wenjun Xiao, Tie Tong, Yongliang Li, Meng Zhang, Xiaoxia Lin, Xiaoxiao Zou, Qun Wu, Xinhong Guo

**Affiliations:** College of Biology, Hunan University, Changsha, 410082 China

**Keywords:** Computational biology and bioinformatics, Evolution, Genetics

## Abstract

DNA barcoding is currently an effective and widely used tool that enables rapid and accurate identification of plant species. The *Orchidaceae* is the second largest family of flowering plants, with more than 700 genera and 20,000 species distributed nearly worldwide. The accurate identification of *Orchids* not only contributes to the safe utilization of these plants, but also it is essential to the protection and utilization of germplasm resources. In this study, the DNA barcoding of 4 chloroplast genes (*matK, rbcL, ndhF* and *ycf1*) were used to provide theoretical basis for species identification, germplasm conservation and innovative utilization of *orchids*. By comparing the nucleotide replacement saturation of the single or combined sequences among the 4 genes, we found that these sequences reached a saturation state and were suitable for phylogenetic relationship analysis. The phylogenetic analyses based on genetic distance indicated that *ndhF* and *ycf1* sequences were competent to identification at genus and species level of *orchids* in a single gene. In the combined sequences, *matK* + *ycf1* and *ndhF* + *ycf1* were qualified for identification at the genera and species levels, suggesting the potential roles of *ndhF, ycf1*, *matK* + *ycf1* and *ndhF* + *ycf1* as candidate barcodes for *orchids*. Based on the SNP sites, candidate genes were used to obtain the specific barcode of *orchid* plant species and generated the corresponding DNA QR code ID card that could be immediately recognized by electronic devices. This study provides innovative research methods for efficient species identification of *orchids*. The standardized and accurate barcode information of *Orchids* is provided for researchers. It lays the foundation for the conservation, evaluation, innovative utilization and protection of *Orchidaceae* germplasm resources.

## Introduction

*Orchidaceae* is the second largest family after *Composite*, and the largest family of monocotyledonous plants^1–3^. More than 700 genera and more than 20,000 species were identified in the family *Orchidaceae*, which account for 8 percent of all flowering plants^1,2,4–7^. *Orchids* mainly distribute in the tropical and subtropical regions of the world, and a few species grow in the temperate regions^2–4,8,9^.

The *Orchidaceae* plants exhibit important ornamental, medicinal, research and ecological value^2,10–12^. Many *Orchidaceae* plants with beautiful flowers and rich fragrance are ornamental plants, such as *Cymbidium*, *Phalaenopsis*, *Cypripedium*^2,12–14^. Numerous species containing active ingredients, like polysaccharides, alkaloids, phenanthrene and dibenzyl also are served as traditional herbal medicines for treatment of the diseases^2,7,10,12,15^. These traits that is able to bring great economic benefits make *Orchidaceae* plants on raising market demand. In the past decades, over-exploitation and habitat destruction by humans caused serious extinction threats to a large number of *Orchidaceae* plants^2,10,15^. Additionally, more and more counterfeit and shoddy *Orchidaceae*-related products emerge. This is not only likely to threaten drug safety, but also caused damage to biodiversity^2,7,11,12^.

Given that, the accurate identification of *Orchidaceae* plants is of great significance for their safe utilization, biodiversity and the protection of genetic resources^2,7,12,16,17^. It is known that traditional identification methods are based on morphological features. Some *Orchidaceae* plants, however, alomst exhibit no morphological differences before flowering, and the orphological features are susceptible to environmental factors^2,7, 16,18^. In addition, there are fewer and fewer experienced experts in morphological identification^2,7,12,16,18–20^. Totally, this makes the accurate identification to be a time consuming and labor intensive job. Therefore, we are badly in need of a rapid, accessible and accurate identification method.

The DNA barcode technology is a novel molecular recognition technology that uses short and standard DNA fragments for species identification^7,16,17,19,21,22^. DNA barcodes were originally utilized to identify microorganisms^23^, but now it is able to quickly and accurately identify species at the level of species with unlimited reasons for development stage, internal morphological diversity, environmental factors and user's professional level^2,7,16,18,22,23^. Thus, the DNA barcoding technology has been rapidly applied in species identification, biosystematics, biodiversity, ecological community evolution, species protection, archaeological sample identification and other aspects^1,7,18,24–27^. Mitochondrial cytochrome oxidase I gene proposed by Hebert et al. in 2003 had been widely used in animal species identification and phylogenetic development^28,29^. However, due to the low mutation rate of mitochondrial DNA, mitochondrial cytochrome oxidase I can not be used in plants^21,23,30,31^. In the past decades, many researchers have made great contributions to the search and application of barcode in plants. Subsequently, many scientists performed a great deal of phylogenetic analyses among numerous families or subfamilies of the *orchid* family based on two plastid genes *matK* or *rbcL*^24,30,32–34^. Many efforts have been made to discover the core barcodes for different land plant taxa, whereas a consensus has not been reached^35,36^. After that, CBOL Plant Working Group compared the performance of seven leading candidate plasome DNA regions (*atpF*-*atpH* interval, *matK* gene, *rbcL* gene, *rpoB* gene, *rpoC1* gene, *psbK-psbI* interval and *trnH-psbA* interval) and recommended the 2-site combination of *rbcL* + *matK* as a plant barcode based on the evaluation of recoverability, sequence quality and species identification level^23^. The generality of medicinal plants species identification were assessed according to *matK* and *rbcL* genes^16,27,37–40^. The molecular taxonomic identification of the *Canarian* oceanic hotspot was studied based on *matK* + *rbcL*^41^. Chen et al. found that *ycf1* showed high identification ability at the species level of rare and protected medicinal plants. The chloroplast gene *ndhF* was found to be able to identify 100% solanum species by Zhang et al.^42,43^. Although DNA barcoding has been widely studied in phylogeny and species identification of *Orchids*, it has not been reported that DNA barcoding genes can be used to develop specific identification segments of different species^2,7,9,16,17,44–48^.

Here, we used four chloroplast gene sequences (*matK*, *rbcL, ndhF* and *ycf1*) and three combined sequences including *matK* + *rbcL*, *matK* + *ycf1, ndhF* + *ycf1* of *Orchidaceae* species to develop unique identification fragments of a certain species of *Orchidaceae* based on phylogenetic analyses and SNP site analyses. Furthermore, the barcode genes were comprehensively analyzed to obtain standard DNA marker fragments of *Orchidaceae.* Therefore, this study provided a novel approach, based on the SNP barcode, to accurately and rapidly identify *Orchidaceae* plants. This technology replenishes traditional methods of identification in *Orchidaceae* plants. This is the first study to report a strategy for developing specific DNA barcodes of *Orchidaceae* plants, laying the foundation for the conservation, evaluation, innovative utilization and protection of *Orchidaceae* germplasm resources.

## Results

### DNA sequences analysis

In this study, the sequences including 3040 *matK* sequences (307 genera, 1900 species), 641 *rbcL* sequences (55 genera, 192 species), 225 *ndhF* sequences (102 species, 29 genera), and 384 *ycf1* sequences (48 genera, 173 species) of *Orchids* were obtained from the NCBI Nucleotide database (https://www.ncbi.nlm.nih.gov/) for further analyses.

After blasting and editing, the consensus length of *matK*, *rbcL, ndhF and ycf1* were 2169 bp, 1524 bp, 2953 bp, 8145 bp respectively, and that of combined sequence including *matK* + *rbcL, matK* + *ycf1, ndhF* + *ycf1* were 3348 bp, 9731 bp, 9701 bp, respectively.

The overall mean nucleotide base frequencies observed for candidate nucleotide sequences and the distribution of the four bases of candidate nucleotide sequences at different coding positions of codons were showed in Table 1. The average number of identical pairs (ii) for candidate nucleotide sequences was showed in Table 2. The account of transitional pairs (si) and transversional pairs (sv) of nucleotide sequences was showed in Table 2. The transitional and transversional of bases in the sequences may be related to the species difference.

**Table 1:** The nucleotide base frequencies analysis of candidate nucleotide sequences in *Orchidaceae* plants.

Sequences	Base content										
	A	T	C	G	GC	AT-1	GC-1	AT-2	GC-2	AT-3	GC-3
*matK*	30.8	37.8	16.4	15.0	31.4	68.5	31.5	68.9	31.2	68.3	31.7
*rbcL*	28.0	29.2	18.4	24.5	42.9	62.7	37.3	53.2	46.7	55.4	44.6
*ndhF*	27.3	39.4	16.1	17.2	33.3	66.9	33.1	64.9	35.1	68.4	31.6
*ycf1*	40.4	30.0	13.9	15.7	29.6	69.3	30.8	71.8	28.2	60.2	29.8

**Table 2:** The analysis of nucleotide pair frequencies of candidate nucleotide sequences of *Orchidaceae* plants.

Sequence	ii				si				sv				R			
	Avg	1st	2nd	3rd	Avg	1st	2nd	3rd	Avg	1st	2nd	3rd	Avg	1st	2nd	3rd
*matK*	1268	431	423	414	49	16	15	18	43	13	15	15	1.1	1.3	1.0	1.2
*rbcL*	1378	462	458	458	29	10	10	9	14	4	5	5	2.1	2.6	1.9	1.0
*ndhF*	1234	410	419	404	38	14	12	12	34	11	10	12	1.1	1.3	1.1	0.9
*ycf1*	4521	1514	1509	1498	205	61	72	72	200	68	62	70	1.0	0.9	1.2	1.0
*matK* + *rbcL*	2835	953	936	946	73	19	32	22	56	15	21	21	1.3	1.3	1.6	1.1
*matK* + *ycf1*	6015	1990	2008	2017	247	94	76	77	239	82	81	75	1.0	1.2	0.9	1.0
*ndhF* + *ycf1*	5718	1905	1914	1899	189	63	61	65	187	61	61	65	1.0	1.0	1.0	1.0

*ii* Identical Pairs, *si* Transitionsal Pairs, *sv* Transversional Pairs, *R* si/sv.

Polymorphism site analysis of the candidate nucleotide sequences revealed in Table 3. Among the single sequence and the combination sequence *rbcL* sequence had the least proportion of mutation sites, accounting for 34.8%, while the conservative sites in the corresponding *rbcL* sequence accounted for 64.7%. The sequence *matK* had the highest proportion of mutation sites (70.2%), and the corresponding *matK* sequence had the lowest proportion of conservative sites (18.9%).

**Table 3:** The analysis of variation of candidate barcode sequences in *Orchidaceae* plants.

Sequence	Conserved site	Variable site	Parsimony-informative site	Sigon site
*matK*	411 (18.9%)	1523 (70.2%)	1275	222
*rbcL*	986 (64.7%)	530 (34.8%)	504	26
*ndhF*	1031 (34.9%)	1790 (60.6%)	1492	297
*ycf1*	3291 (40.4%)	4732 (58.1%)	4578	154
*matK* + *rbcL*	1856 (55.4%)	1455 (43.5%)	1369	86
*matK* + *ndhF*	2017 (44.5%)	2377 (52.4%)	2027	348
*matK* + *ycf1*	3996 (41.1%)	5506 (56.6%)	5247	259
*ndhF* + *ycf1*	4217 (43.5%)	5299 (54.6%)	4696	592

### Genetic diversity

There must be some genetic variation based on their species differences since the data used to analyze were obtained from different species. The basic indicators of genetic diversity, displayed in Table 4, worked out in accordance with pairwise nucleotide differences and nucleotide diversity, and the validity of these indexes were verified by two neutrality tests, like Fu’s *Fs*^49^ and Tajima’s *D*^50^. The *matK* + *ycf1* sequences had revealed maximum genetic diversity cumulatively on the base of Eta value, revealed 2314 mutations within all sequences. While the *rbcL* sequences only had 322 mutations variations in all sequences. The significance of genetic diversity was verified by both neutrality tests, which confirmed that all sequences had significant difference but no very significant difference based on the probability value (p-value) of Fu’s *Fs* test and Tajima’s *D* test (Table 4).

**Table 4:** Genetic diversity caculation of *Orchidaceae* plants based on candidate barcode sequences by the DnaSP v5 software.

Sequences	n	Nucleotide diversity					π	Neutrality tests			
		S	k	Eta	Hd	θ		Fu’s *Fs*	p-value	D	p-value
*matK*	3050	1288	3.12596	1523	0.9050	0.24339	0.07270	− 2.57476	< 0.05	− 1.84286	< 0.05
*rbcL*	643	259	15.997	322	0.9779	0.07155	0.02503	− 0.51015	> 0.10	− 1.92689	< 0.05
*ndhF*	234	233	13.952	340	0.9660	0.18546	0.04589	− 2.96843	< 0.05	− 2.37565	< 0.01
*ycf1*	384	906	95.110	1470	0.9921	0.17379	0.07339	0.25100	> 0.10	− 1.78584	< 0.05
*matK* + *rbcL*	372	559	54.729	821	0.9924	0.12616	0.05462	− 0.11121	> 0.10	− 1.75132	< 0.05
*matK* + *ndhF*	216	687	59.444	943	0.9853	0.12276	0.04604	− 1.32301	> 0.10	− 2.00131	< 0.05
*matK* + *ycf1*	378	1495	150.984	2314	0.9932	0.15463	0.06542	0.08057	> 0.10	− 1.79023	< 0.05
*ndhF* + *ycf1*	228	494	39.916	712	0.9740	0.15585	0.05191	− 1.47948	> 0.10	− 2.13392	< 0.01

*Eta* Total number of mutations, *n* number of sequences, *k* Average number of nucleotide difference, *S* Number of segregating sites, *θ* nucleotide substitution rate, *π* nucleotide diversity, *Hd* haplotype diversity, *Fu’s* *Fs* is variation among different haplotypes in the population, *D* is the Tajima test statistic.

**Figure 1:**
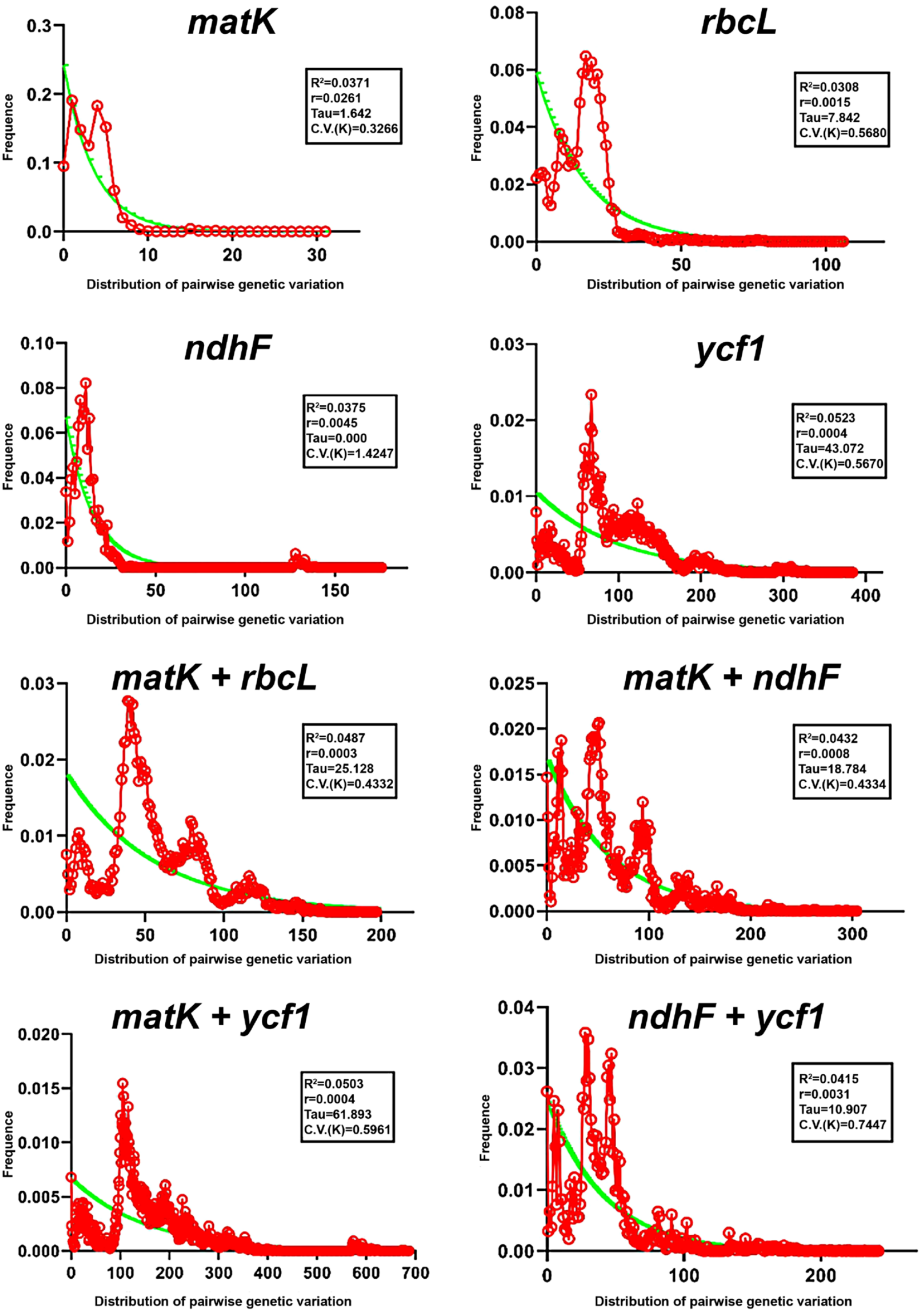
Pairwise mismatch distributions, based on *matK*, *rbcL*, *ndhF*, *ycf1* and the combined sequences by DnaSP v5. Note: The X-axis shows the observed distribution of pairwise genetic variation, and the Y-axis shows the frequency. *R*^*2*^ Ramos-Onsins and Rozas statistics, *r* Raggedness statistic, *Tau *Date of the Growth or Decline measured of mutational time, *C.V. *Coefficient of variation.

Like the neutrality testes of the Tajima test statistic (D value) in the sequences, the genetic variation for *ndhF* sequences was negatively little higher (− 2.37565) with respect to *rbcL* sequence, consisting value up to − 0.51015. And for combined sequences, the genetic variation for *ndhF* + *ycf1* sequences was negatively little higher (− 2.13392) with respect to *matK* + *rbcL* sequence, consisting value up to − 1.75132. With respect to Fu’s *Fs* value for sequences variation, the *ndhF* sequences was higher sequences variation (− 2.96843), shown in Table 4, in comparison with *rbcL* sequence (− 0.51015). In order to observe nucleotide mismatch distribution among different sequences of *Orchidaceae* species, DNA sequences were analyzed for population size changes which was enriched the results of genetic diversity among species. All results showed significant genetic variation in *Orchidaceae* species for candidate nucleotide sequences (Fig. 1).

### Phylogenetic analysis

**Figure 2:**
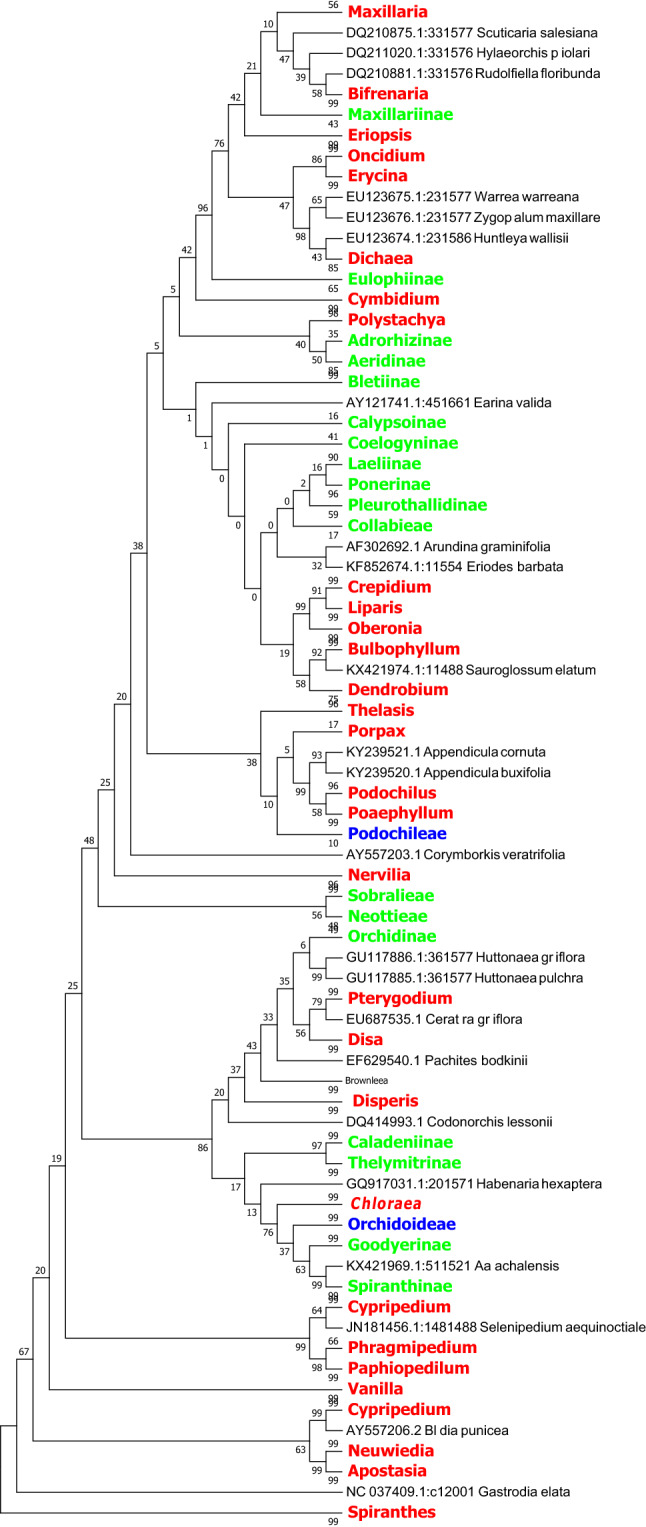
The NJ tree of *Orchidaceae* coming from analysis of the cp DNA *matK* sequence based on the K2P model. Names tagged in red indicates the genus, tagged in green showed the subtribe and tagged in blue showed the subfamily; The Numbers on the branches represent more than or equal to 50 percent support after the 1000 bootstrap replications test; Numbers following taxon names showed the number of species.

**Figure 3:**
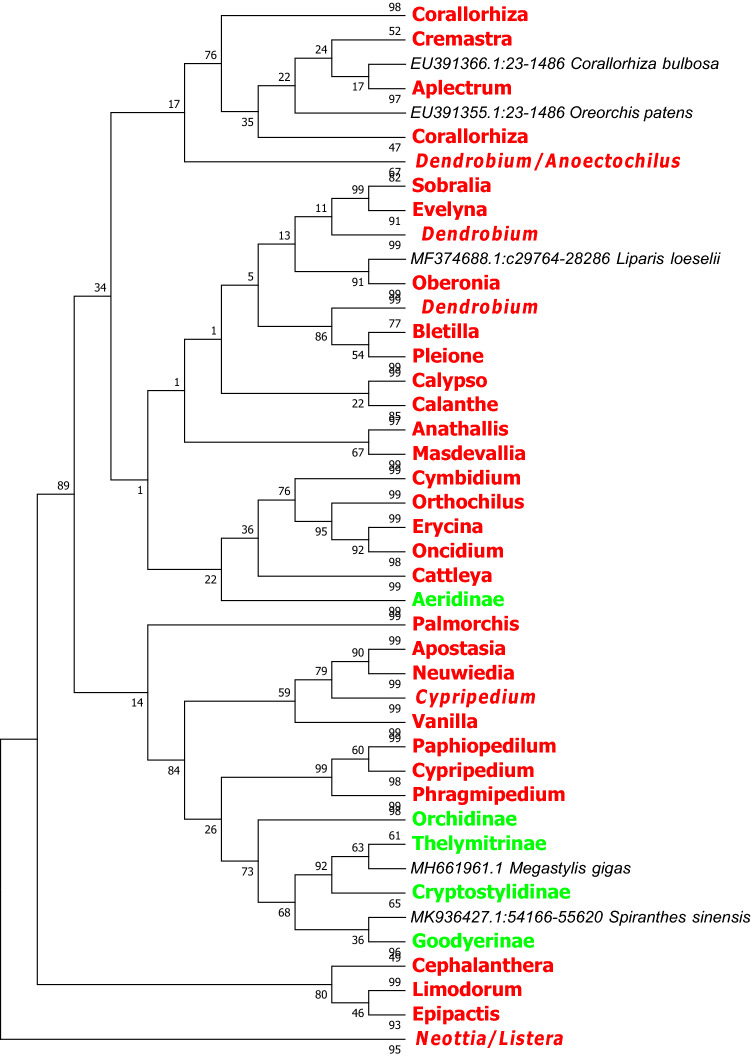
The NJ tree of *Orchidaceae* coming from analysis of the cp DNA *rbcL* sequence based on the K2P model. Names tagged in red indicates the genus and tagged in green showed the subtribe; The Numbers on the branches represent more than or equal to 50 percent support after the 1000 bootstrap replications test; Numbers following taxon names showed the number of species.

**Figure 4:**
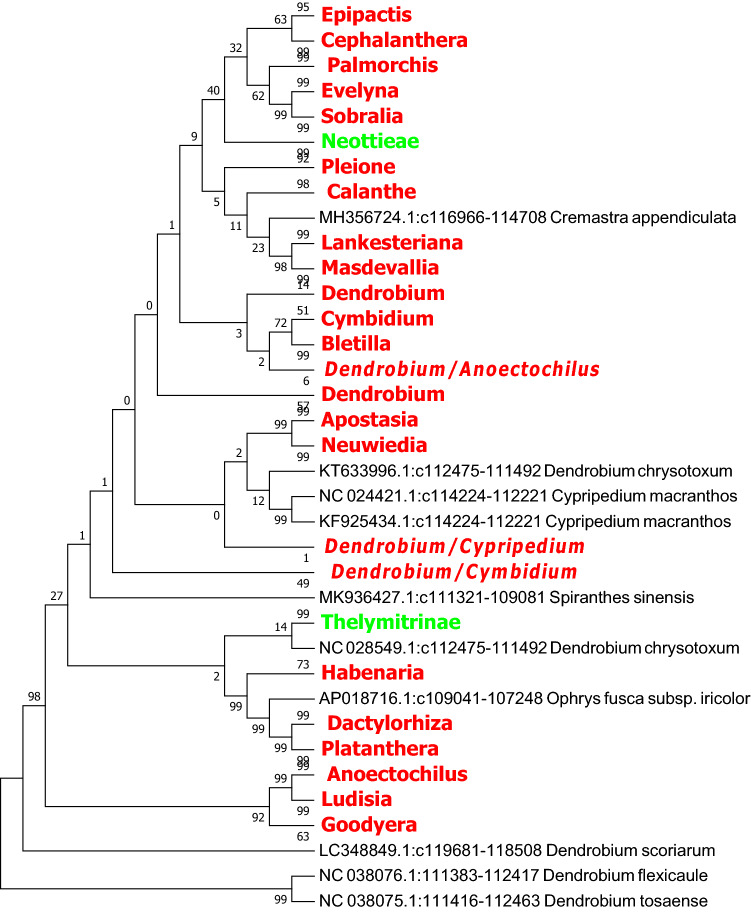
The NJ tree of *Orchidaceae* coming from analysis of the cp DNA *ndhF* sequence based on the K2P model. Names tagged in red indicates the genus and tagged in green showed the subtribe; The Numbers on the branches represent more than or equal to 50 percent support after the 1000 bootstrap replications test; Numbers following taxon names showed the number of species.

**Figure 5:**
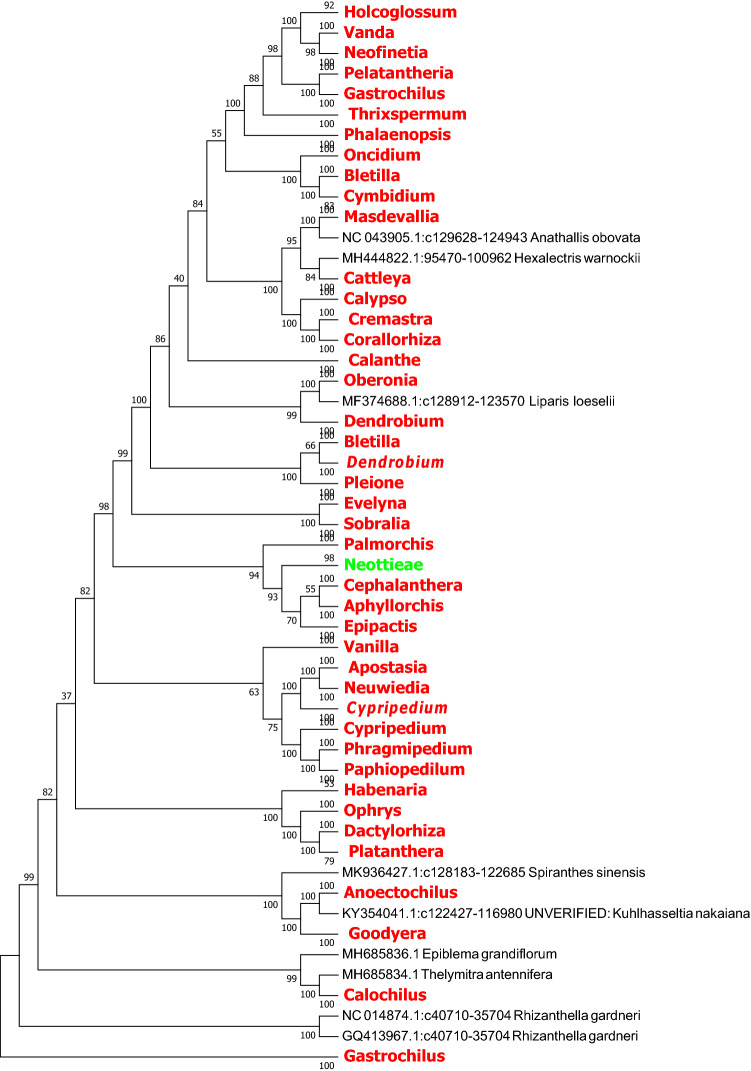
The NJ tree of *Orchidaceae* coming from analysis of the cp DNA *ycf1* sequence based on the K2P model. Names tagged in red indicates the genus and tagged in green showed the subtribe; The Numbers on the branches represent more than or equal to 50 percent support after the 1000 bootstrap replications test; Numbers following taxon names showed the number of species.

**Figure 6:**
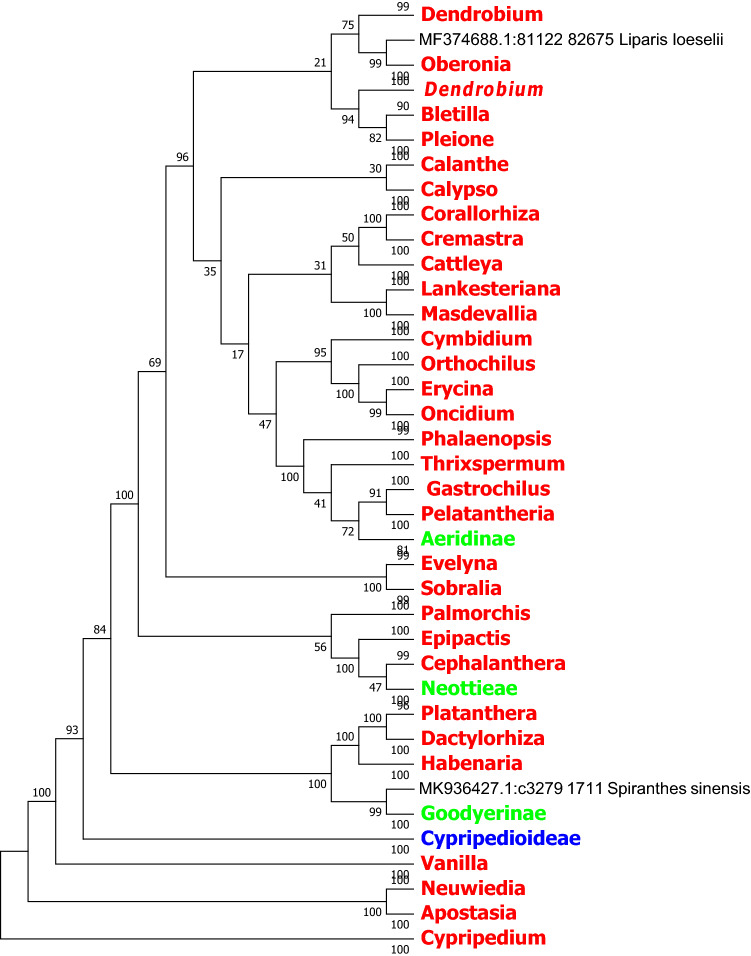
The NJ tree of *Orchidaceae* coming from analysis of the *matK* + *rbcL* sequence based on the K2P model. Names tagged in red indicates the genus and tagged in green showed the subtribe. The Numbers on the branches represent more than or equal to 50 percent support after the 1000 bootstrap replications test. The Numbers following taxon names showed the number of species.

**Figure 7:**
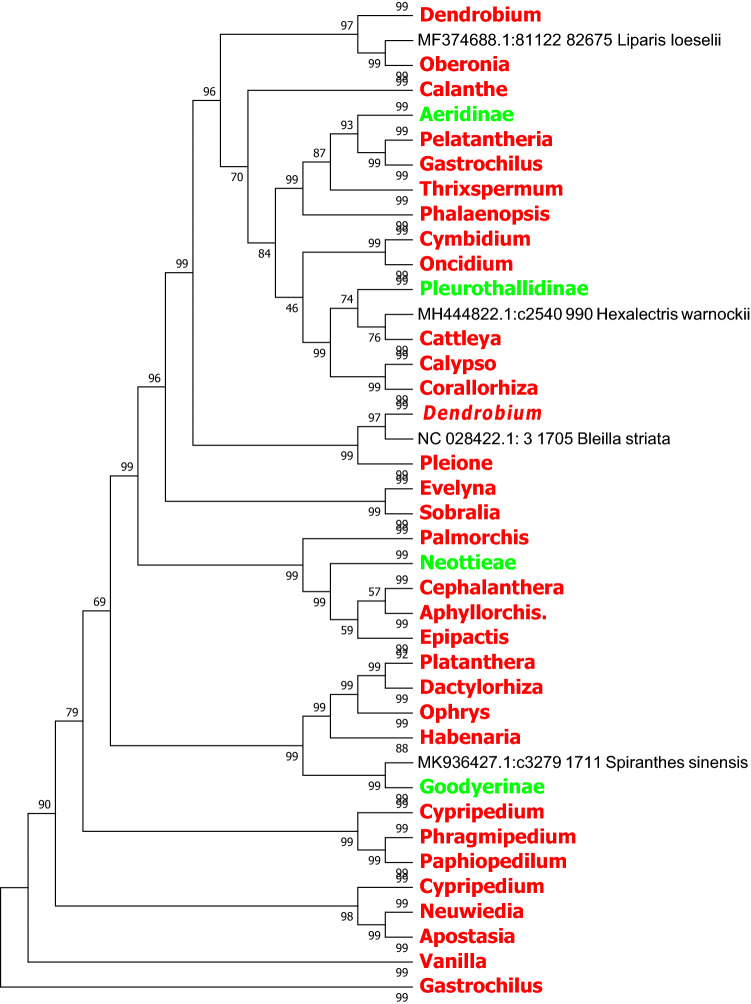
The NJ tree of *Orchidaceae* from analysis of the *matK* + *ycf1* sequence based on the K2P model. Names tagged in red indicates the genus and tagged in green showed the subtribe; The Numbers on the branches represent more than or equal to 50 percent support after the 1000 bootstrap replications test; Numbers following taxon names showed the number of species.

**Figure 8:**
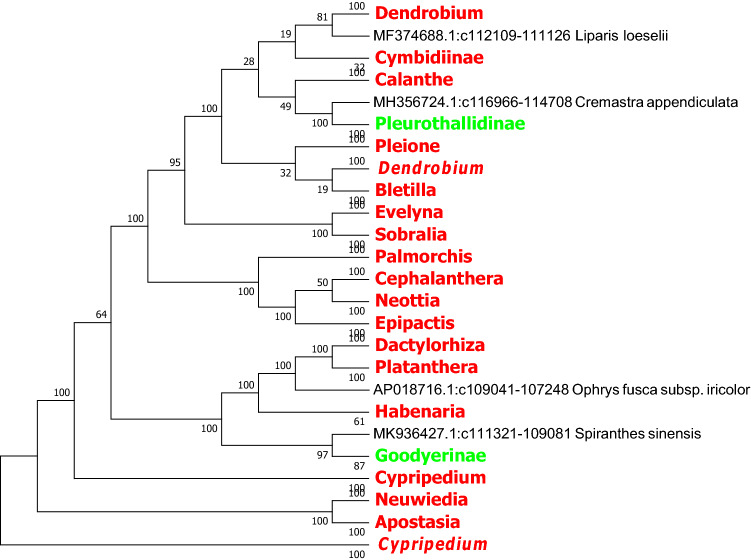
The NJ tree of *Orchidaceae* from analysis of the *ndhF* + *ycf1* sequence based on the K2P model. Names tagged in red indicates the genus and tagged in green showed the subtribe.

In this study, we used the MEGA7.0 software based on the Neighbor Joining method and Kimura 2-parameter model to identify *rbcL, ndhF* and *ycf1* sequence of the evolutionary tree, and we compressed the same genera or the same subtribes of *Orchid* with the MEGA 7.0 own Compress Subtree. In the light of the topological structure of the evolutionary tree, species in several subfamilies are not be well identified based on *matK*, *rbcL* and the combined sequence *matK* + *rbcL*. In contrast, the *ndhF*, *ycf1* sequences and the combined sequences *matK* + *ycf1* and *ndhF* + *ycf1* of chloroplast genes exhibit better identification ability at the generic level (Figs. 2, 3, 4, 5, 6, 7, 8).

### Analysis of barcoding gap

**Figure 9:**
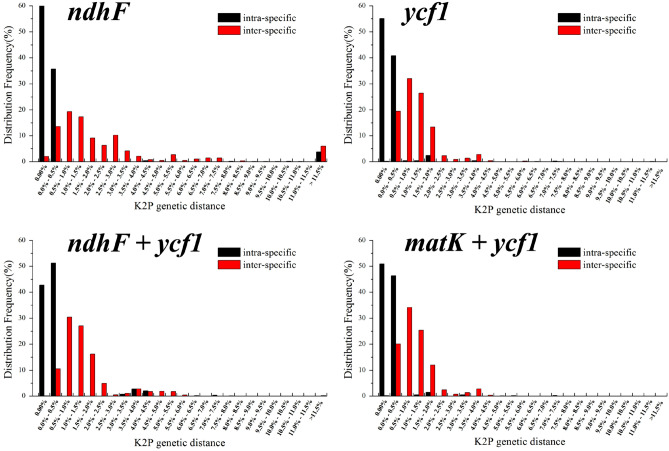
Histogram of frequency of intra-species (black) and inter-species (red) of *Orchidaceae* based on K2P distance of candidate genes. The X-axis represents the genetic distance, and the Y-axis represents the frequency.

An ideal DNA barcoding sequence for species identification should satisfy that inter-specific genetic variation is significantly greater than intra-specific genetic variation. In order to more accurately assess individual chloroplast genes and combined sequences in the *Orchid* genus species, and to verify the applicability of candidate sequences, the barcoding gap was analyzed according to frequency distribution showed in Fig. 9. The results revealed that the *ndhF* gene showed better performance in a single gene, while the combined sequences of *ndhF* + *ycf1* showed the best performance. The results of the Best Close Match of several candidate barcodes based on genetic distance are showed in Table 5. Among the single genes, the accuracy rate of *ycf1* gene for *orchid* plant identification is 89.32%, with 3.38% fuzzy identification rate and 6.25% error identification rate. The *ndhF* gene exhibits the highest identification rate and lower error rate of *matK* + *ycf1*, followed by *ndhF* + *ycf1* sequence. The accuracy of *matK* + *ycf1* sequence was 89.6%, with 2.8% fuzzy identification rate and 1.12% error identification rate. The accuracy rate of *ndhF* + *ycf1* sequence was 88.78%, with 2.33% fuzzy identification rate and 2.8% error identification rate. The data indicated that *ndhF* and *ycf1* were suitable for the identification of *Orchids* at the level of genus and species, while the combined sequences of *matK* + *ycf1* and *ndhF* + *ycf1* were qualified at the genera and species levels.

**Table 5:** Best Close Match test results based on genetic distance.

Sequences	Correct	Fuzzy	Error	Did not identify
*ndhF*	88.65%	3.45%	2.50%	5.48%
*ycf1*	89.32%	3.38%	6.25%	1.05%
*matK* + *ycf1*	89.60%	2.80%	1.12%	6.48%
*ndhF* + *ycf1*	88.78%	2.33%	2.80%	6.09%

### Specific barcodes based on SNP sites

**Figure 10:**
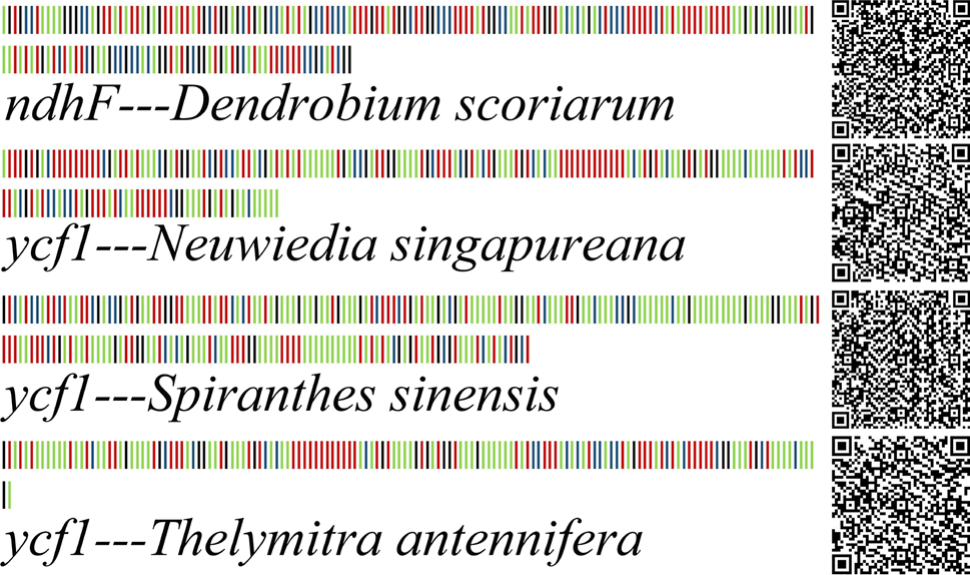
DNA barcodes and two-dimensional DNA barcodes of *Orchidaceae* species based on *ndhF* and *ycf1* genes. Base A in green, base T in red, base C in blue, and base G in black.

**Figure 11:**
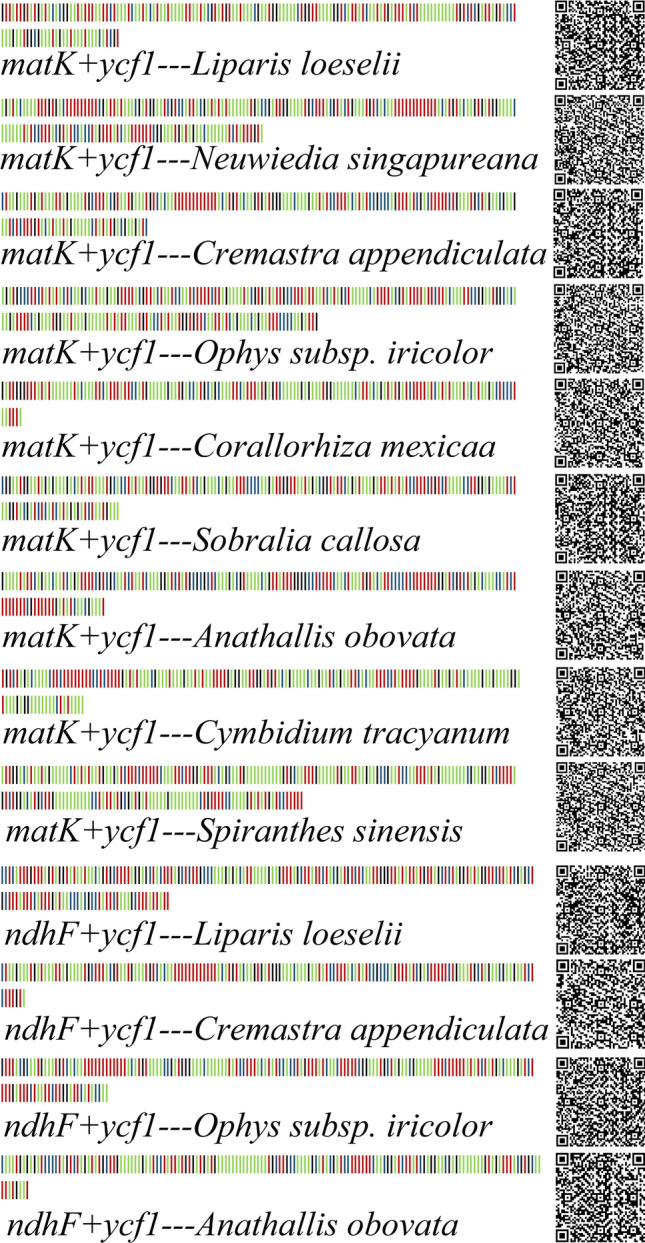
DNA barcodes and two-dimensional DNA barcodes of *Orchidaceae* species based on *matK* + *ycf1* and *ndhF* + *ycf1* genes. Base A in green, base T in red, base C in blue, and base G in black.

Based on SNP sites, species-specific barcodes were developed and the appropriate fragments were blasted into the NCBI database. Based on the *ndhF* sequence, the specific barcode of species *Dendrobium scoriarum* was obtained. Knowledge about specific barcodes of species *Neuwiedia thelymitra*, *Spiranthes sinensis* and *Epiblema cocflorum* based on *ycf1* sequence was obtained. Based on the combined sequence *ndhF* + *ycf1*, the specific barcodes of *Liparis loeselii*, *Cremastraa ppendiculata*, *Spiranthes siensis* and *Anathallis obovata* were obtained, whereas *Liparis loeselii* and *Cremastra appendiculata* had two specific barcodes. Two-Dimensional code can be scanned by electronic equipment from DNA fragments that can be used for species identification. It can provide theoretical support for subsequent researchers. Using the Two-Dimensional code coding method, the species-specific barcode obtained was converted into two-dimensional barcode image, which was conducive to the conversion of barcode information (Figs. 10, 11).

## Discussion

DNA barcode is able to be utilized for species identification by means of a DNA fragment that is common to all species. The fragment must simultaneously contain adequate variability to allow for species identification and enough conservative area for the design of universal primers^21,23^. So far, DNA barcoding have been widely used in many genera of *Orchidaceae*^1,2,7,16^. As far as we know, it is the first time that multi-aspect analysis in species identification of *Orchidaceae* with such a well-rounded species size, based on *matK* and *rbcL* regions.

The results of sequences analyses on average GC content showed that the GC content of candidate sequences of *Orchids* was far less than AT content, while significantly less than the GC content of about 50% in common angiosperms. Of sequence variation situation analysis, the candidate gene mutations exist base insert and missing phenomenon. We performed the analysis of the genetic diversity by the DnasP 5.0 software. The higher haploid type diversity and relatively low haploid type diversity of nucleotide diversity demonstrated that the candidate sequences had certain polymorphisms.

The CBOL recommends *matK* and *rbcL* as universal barcodes in plant kingdom^23^. With the development of science and technology, many subsequent scientists have evaluated the discriminability of different DNA barcoding genes in different families or genera, but the discriminability of a candidate gene in different plants was different.

On the basis of phylogenetic relationship, the Barcoding Gap and the Best Close Match with the genetic distance in evaluating candidate barcode identification capability in *Orchid*, the phylogenetic analyses showed that the identification ability of *matK* and *rbcL* was low on the genus level. The possible reason was that there were more species in this study, which made the species in the related genus unable to form branches alone. The sequences of *ndhF* and *ycf1* were suitable for identification of genus and species of *Orchids*, and the combined sequences *matK* + *ycf1* and *ndhF* + *ycf1* were qualified at the genera and species levels. The Barcoding Gap test indicated that these candidate genes all contained Barcoding Gap, and the variation between species and within species had clear boundaries. The test results of Best Close Match revealed that the all combined sequences exhibited high genus identification rate, which was suitable for the identification of orchids at the level of genus and species.

Based on the SNP sites, the species level specific DNA barcodes of *Orchid* were successfully developed. Combinatorial sequences were able to develop more species-specific barcodes than chloroplast genes, which might be the result of combination sequences could provide more mutation sites and SNP sites. There were some differences in the specificities of different combination genes in *Orchidaceae* plants. Compared with *ndhF* + *ycf1*, the combined sequences of *matK* + *ycf1* could be developed more specific barcodes, which might be related to the species identification accuracy of *matK* + *ycf1* in *Orchids.*

## Conclusion

In summary, *ndhF*, *ycf1*, *matK* + *ycf1* and *ndhF* + *ycf1* sequences are competent to develop species-specific barcodes to identify *Orchidaceae* plants at the molecular level. Cluster analysis using the *ndhF*, *ycf1*, *matK* + *ycf1* and *ndhF* + *ycf1* sequences in *Orchid* are nearly consistent with traditional plant morphology. Additionally, this study not only broadens the application of the *matK* and *rbcL* sequences in the barcode field, but also provides a novel thought to expand species identification method in a wide range of plant at the species level.

## Methods

### Nucleotide sequences

For species identification, we retrieved the chloroplast DNA reference sequences including *matK*, *rbcL, ndhF and ycf1* from the NCBI Gene database (https://www.ncbi.nlm.nih.gov/). We obtained the combined sequence including *matK* + *rbcL*, *matK* + *ycf1, ndhF* + *ycf1* by supermat's function in R Phylotools package. After manual screening, the short nucleotide sequences were deleted, and the sequences with different directions were modified manually.

### Data analysis

We performed the sequences alignment by the Muscle in the MEGA 7.0 software^51^ (https://www.megasoftware.net/) with the default alignment parameters for multiple sequences alignment parameters. In the pairwise distances analyses, the positions containing gaps and missing were eliminated from the data set (complete deletion option). Phylogenetic trees constructed with the Neighbor-joining (NJ) method according to Kimura 2-Parameter (K2P) model was assessed by the MEGA 7.0^9,46,52^. The clade reliability in these trees using the NJ methods was tested by bootstrapping, in which 1000 repeated sampling tests were performed to obtain the support values of the clade nodes. Polymorphic site, genetic diversity indices and neutrality tests [Fu’s *Fs*^49^ and Tajima’s *D*^50^] were performed by the DnaSP v5^53^ (http://www.ub.edu/dnasp/index_v5.html).
